# Roles of Supersaturation and Liquid–Liquid Phase Separation for Enhanced Oral Absorption of Poorly Soluble Drugs from Amorphous Solid Dispersions

**DOI:** 10.3390/pharmaceutics17020262

**Published:** 2025-02-16

**Authors:** Kohsaku Kawakami

**Affiliations:** 1 Research Center for Macromolecules and Biomaterials, National Institute for Materials Science, 1-1 Namiki, Tsukuba 305-0044, Ibaraki, Japan; kawakami.kohsaku@nims.go.jp; 2 Graduate School of Science and Technology, University of Tsukuba, 1-1-1 Tennodai, Tsukuba 305-8577, Ibaraki, Japan

**Keywords:** amorphous solid dispersion, crystallization, supersaturation, liquid–liquid phase separation, membrane permeability, oral absorption

## Abstract

Amorphous solid dispersion (ASD) is one of the most important enabling formulation technologies for the development of poorly soluble drugs. Because of its thermodynamically unstable nature in both solid and wet states, the evaluation and optimization of the formulation performance involves some difficulties. The dissolution process is sensitively influenced by various factors, including the applied dose, medium composition, and pH. Supersaturated solutions can cause liquid–liquid phase separation (LLPS) and/or crystallization, which complicates the comprehension of the dissolution process. However, LLPS should be evaluated carefully because it is closely related to oral absorption. As LLPS concentration is analogous to amorphous solubility, it can be a key factor in predicting oral absorption from ASDs, if absorption is limited by solubility. Moreover, LLPS droplets are expected to increase transmembrane flux by increasing the drug concentration near the epithelial cell membrane. In this review, recently updated knowledge on the dissolution, membrane permeation, and oral absorption behaviors of ASDs is discussed with an emphasis on LLPS behavior.

## 1. Introduction

Amorphous solid dispersion (ASD) is widely recognized as a powerful formulation technology for poorly soluble drugs. Its usage is accelerating year by year. Between 2012 and 2023, 48 drug products that contain ASD have been approved by the FDA [1]. The amorphous state offers higher solubility than that of the crystalline state [2,3]; therefore, oral absorption of a drug may be enhanced if it is limited by solubility or dissolution rate [4,5,6,7,8]. As solutions in the supersaturated state have solute concentrations that exceed equilibrium solubility, those solutions are not thermodynamically stable. Therefore, an appropriate formulation design is required for ASDs to effectively maintain supersaturation. For the crystalline solids, the dissolved drug concentration keeps increasing until the equilibrium state is achieved, whereas, for amorphous solids, the dissolution profile may decrease after reaching maximum concentrations under non-sink conditions that have been described as the “spring and parachute” [4]. The decrease in concentration is induced either by crystallization or liquid–liquid phase separation (LLPS) [9,10,11]. Understanding what happens during the dissolution process of ASDs is crucial for rational formulation design. The advantage of ASD is not expected if crystallization proceeds promptly during dissolution. If LLPS occurs, the activity of the solution is different from that of a “real” supersaturated solution, where all drug molecules are dissolved as solutes. A dissolution test is the first step in evaluating the potential of the formulations. However, both the selection of the experimental condition and the interpretation of the results are still quite challenging for ASDs. In this review, the dissolution process of ASDs is discussed with an emphasis on the dissolved state of drug molecules and its impact on membrane permeation and oral absorption behavior.

## 2. Supersaturation and LLPS

**Figure 1:**
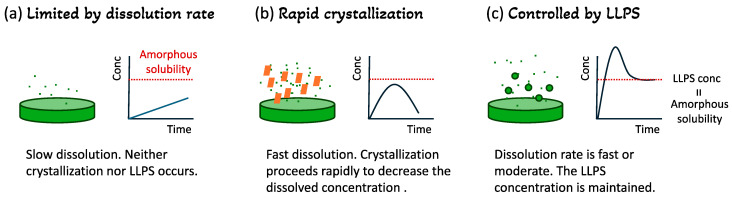
Typical patterns of ASD dissolution profiles. (**a**) Drug release is limited by dissolution. Small dots represent released drug molecules. Neither crystallization nor LLPS occurs in the solution. Direct transformation from amorphous to crystal in the solid can proceed. (**b**) Supersaturation is immediately destroyed if crystals appear. The crystals that appeared from the supersaturated solution are indicated by the orange squares. Crystallization can also happen on solid surface. (**c**) If the dissolution is sufficiently rapid to induce LLPS and the state is stable, the LLPS concentration, which is analogous to amorphous solubility, is maintained. The green circles represent LLPS droplets.

Supersaturation and LLPS are important events for ASDs to improve oral absorption of poorly soluble drugs, which must be appropriately evaluated for their development. Both events can be observed only under non-sink conditions in the dissolution test. When an amorphous drug or ASD is dissolved in a medium under the non-sink condition, three major scenarios are available in the dissolution process (Figure 1). (1) If the dissolution is slow, the drug concentration may not reach amorphous solubility. In this case, further formulation efforts are required to accelerate the dissolution rate to achieve supersaturation. (2) If the dissolution is too rapid and/or the crystallization tendency of the drug is too high, and unless the stabilization effect of the polymer is sufficient, crystallization may proceed after supersaturation is achieved. Such an ASD may work for drugs with high permeability if absorption proceeds before crystallization occurs. However, efforts to retard crystallization are generally preferred. (3) LLPS may occur after “real” supersaturation and continue for a long time under appropriate formulation design. This is the most favored for enhancing the oral absorption of poorly soluble drugs, as high drug concentration is expected to be maintained in the gastrointestinal tract for a long duration. When crystals appear, supersaturation/LLPS is destroyed. However, it may not happen immediately after the appearance of crystals [12]. The dissolution behavior of ASDs depends on both formulation properties and dissolution conditions. Thus, in addition to the appropriate design of ASDs, the appropriate design of test conditions, which requires an understanding of the environment of the gastrointestinal tract, is crucial for the successful evaluation and development of ASDs.

**Figure 2:**
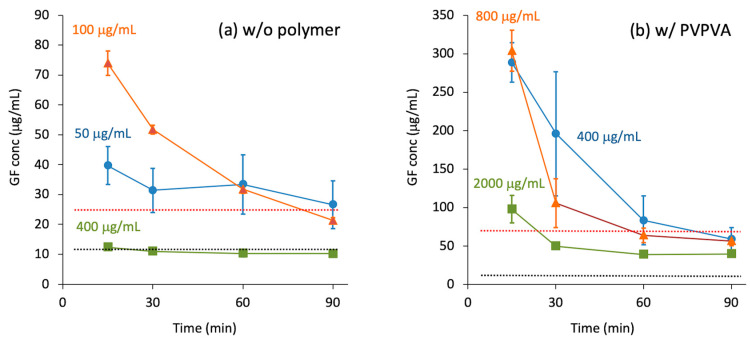
Concentration–time curves of supersaturated GF solutions (phosphate buffer, pH7.0) in the absence (**a**) and presence of PVPVA (**b**) [13]. The supersaturation was created by adding concentrated acetone solution of GF to the buffer. The concentrations presented in the figure represent initial GF concentration. The red and black dotted lines are apparent LLPS concentration and crystalline solubility of GF, respectively. Figures are adopted from ref. [13] after modifications with permission from Elsevier.

Supersaturation achieved by dissolution of amorphous solids can be understood using the basic thermodynamic equation:

(1) ΔG=RTlnx,

Here, Δ*G* and *x* are the Gibbs energy of dissolution and mole fraction, respectively. *R* and *T* are the gas constant and temperature, respectively. As the amorphous state possesses a higher energy state relative to the crystals, a larger *x* is expected, which is analogous to amorphous solubility. However, the rapid dissolution of amorphous solids may offer a higher concentration than the amorphous solubility, that is, “real” supersaturation, which results in crystallization or LLPS [9]. The crystallization rate depends on the degree of supersaturation, the crystallization tendency of the drug, and the stabilization effect of the excipients. Figure 2a shows the precipitation/LLPS behavior of supersaturated griseofulvin (GF) solutions [13]. When the degree of supersaturation was marginal, the concentration decreased slowly until reaching the LLPS concentration. As the degree of supersaturation increased, the concentration decreased more rapidly over time. When the degree of supersaturation was sufficiently high, crystallization proceeded quickly to reach crystalline solubility, which was much lower than the LLPS concentration. Consequently, the order of the dissolved concentration in the final state was completely opposite to that of the initial concentration. In the presence of vinylpyrrolidone-vinyl acetate copolymer (PVPVA) (Figure 2b), supersaturation was effectively maintained relative to the solutions without the polymer. Nevertheless, the trend in reaching the LLPS concentration was the same; that is, the decrease in concentration was faster for solutions with a higher degree of supersaturation.

**Figure 3:**
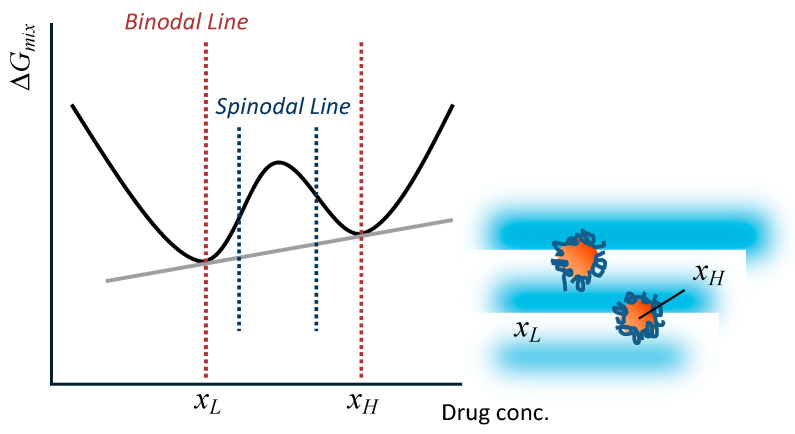
Schematic representation of LLPS.

When LLPS occurs, assuming that the supersaturated solution is separated into concentrated and diluted phases with concentrations of *x*_H_ and *x*_L_, the Gibbs energy of mixing, Δ*G*_mix_, can be calculated as follows:

(2) $$\Delta G_{mix}=f_{H}RTlnx_{H}+f_{L}RTlnx_{L},$$

where *f*_H_ and *f*_L_ are the fractions of concentrated and diluted phases, respectively. This process is illustrated in Figure 3. When phase separation occurs, the sum of the Gibbs energies of each phase falls on the gray tangent line in the figure and is, therefore, lower than the Gibbs energy without phase separation. When the drug concentration is between two spinodal lines (i.e., inflection points), phase separation occurs via spinodal decomposition. The concentration between the binodal and spinodal lines results in phase separation based on the nucleation/growth of the concentrated phase (this does not mean the crystallization of drug molecules). The final composition after the phase separation does not depend on the phase separation mechanism.

When LLPS occurs after the dissolution of ASDs, a colloidal structure is obtained that cannot be explained solely by the thermodynamics mentioned above. The formation of dispersed LLPS droplets increases the Gibbs energy relative to the separation into two distinct phases because of the increase in interfacial energy. Small LLPS droplets with sizes in the order of hundreds of nanometers tend to be formed in the presence of charged polymers, such as Eudragit and hydroxypropylmethylcellulose acetate succinate (HPMCAS) [14,15,16,17]. The decrease in the interfacial tension due to polymer adsorption and electric/steric repulsion between the droplets are responsible for the stabilization of the small droplets. Thus, in the presence of neutral polymers or in the absence of polymers, the size of LLPS droplets is typically larger than 1 μm [13,15].

The LLPS concentration (*x*_L_) can be an important factor in determining transmembrane flux and oral absorption, as described later. Table 1 summarizes examples of the LLPS concentrations for various drugs. In most cases, the LLPS concentration is higher than the crystalline solubility by more than one order of magnitude.

The presence of a small amount of polymer does not influence the LLPS concentration in most cases. However, under competition of crystallization and supersaturation, the LLPS concentration can appear differently. HPMCAS is frequently reported to offer high apparent LLPS concentration [24] because of the strong ability of cellulose polymers to inhibit crystallization [19,25]. Although this concentration does not have thermodynamic significance, it is of practical importance for determining oral absorption. The polymers may participate in the formation of nanodroplets. In particular, cellulose polymers are likely to be preferentially distributed to the colloidal phase [16,19]. Moreover, polymers may independently solubilize the drug, as it is reported to occur with methacrylate polymers (Eudragit) [16].

Surfactants can also be included both in formulations and dissolution medium. If surfactant concentration is above critical micellar concentration, poorly soluble drugs are dissolved by micelles to increase the apparent LLPS concentration [15]. However, its effect on the supersaturation/LLPS behavior is complicated, as the presence of surfactants accelerates both dissolution and crystallization rates.

## 3. Dissolution of ASDs

**Figure 4:**
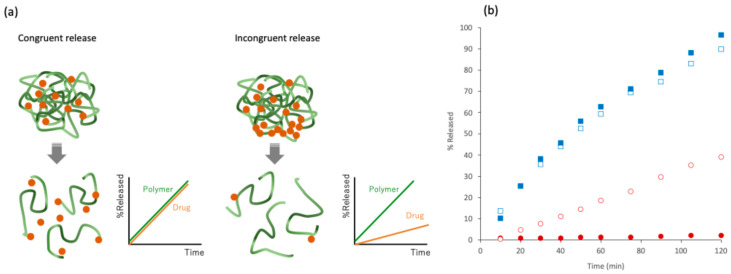
(**a**) Schematic representation of congruent and incongruent release from ASDs. (**b**) Dissolution test of nilvadipine/PVPVA ASDs. The ratio of nilvadipine/PVPVA is 10/90 (blue) or 15/85 (red). Nilvadipine and PVPVA are shown as closed and open symbols, respectively. Data are taken from ref. [26] with permission from Elsevier.

ASDs typically contain hydrophilic polymers to improve the dissolution behavior and storage stability of amorphous drugs. A small amount of surfactant may also be added to improve stability and dissolution behaviors. Although a larger drug/polymer ratio is favored from the viewpoint of pill burden, a sufficient amount of polymer is required to ensure congruent drug release and physical stability of the drug. Unless the amount of polymer is sufficient to achieve a congruent release, the polymer is preferentially dissolved first, leaving the amorphous drugs concentrated in the formulation, which results in the slow dissolution of the drug (Figure 4a). Saboo et al. reported that the release of nilvadipine [26] (Figure 4b) or indomethacin [27] from ASDs formulated with PVPVA proceeded with a congruent release mechanism only when the drug amount was lower than 10%. Ueda et al. investigated the release of nifedipine loaded at a concentration of 25% with a mixture of HPMC and Eudragit S occurred via congruent release only when the Eudragit/HPMC ratio was higher than 50% [28]. Only the drug concentration has been evaluated in most dissolution studies of ASDs reported. However, the measurement of the polymer concentration is also required to judge whether the dissolution is based on congruent or incongruent release. From this aspect, the upper limit of drug loading is called the limit of congruency (LoC) and is likely to be correlated with the strength of the interaction between the drug and the polymer [29]. However, the above discussion concerns binary or trinary mixtures composed only of drugs and polymers. Dissolution behavior is influenced by the addition of disintegrants, including inorganic and effervescing salts [30,31,32]. Also, the deposition of highly soluble components on the surface of ASD may work to improve the dissolution behavior [33]. These efforts may improve the congruency of the dissolution process of ASDs.

To reduce formulation size, great attention has recently been paid to the design of ASDs with high drug loading. One interesting approach is to include drug-rich nanoparticles in ASDs to facilitate the formation of LLPS droplets during the dissolution process [34,35]. In this attempt, the LLPS droplet size was determined beforehand and then nanoparticles of identical sizes were dispersed in the ASD. This approach may be valid for avoiding the incongruent release problem mentioned above, although it should only be applicable to drugs with low crystallization tendency. As drug molecules are locally concentrated in the formulation, unlike typical ASDs where drug and polymers are expected to be mixed at a molecular level, the physical stability of the ASD should be directly influenced by crystallization tendency of drug.

The dissolution test is the simplest method for predicting oral absorption; however, the testing procedure and interpretation of the results for supersaturable formulations are not straightforward [36]. Moreover, the dissolution tests of supersaturable formulations frequently suffer from reproducibility issues [37], which should also be true for ASDs. Box 1 summarizes the points to consider when performing in vivo predictable dissolution tests for ASDs. It should be stressed that the discussion below is not for quality control purposes [3] but is aimed at predicting oral absorption. The use of a non-sink condition is inevitable, as supersaturation must be investigated for prediction. Conventional dissolution tests under sink conditions only provide dissolution rates that are not necessarily correlated with in vivo absorption [38,39]. As the supersaturation behavior is sensitive to the degree of supersaturation, as shown in Figure 2, the selection of the applied dose is also important. “Sink index (SI)” is a convenient parameter for describing the degree of the sink conditions, which can be defined as

(3) $$SI=\frac{C_{s}}{Dose/V},$$

where *C*_s_, *Dose*, and *V* represent the crystalline solubility, applied dose, and medium volume, respectively [40,41]. The appropriate SI range for the in vivo predictive dissolution test depends on the compound. For drugs with solubilities higher than 1 μg/mL, the SI of FDA-recommended or USP dissolution methods for selected ASD products ranges from 0.005 to 0.7, with some exceptions [41]. In our experience, SI values from 0.01 to 0.06 offered a good correlation with in vivo absorption [13,17,22], which corresponds to a slightly higher concentration relative to LLPS.

Box 1 **Points to be considered for in vivo predictable dissolution test for ASDs.**

✓ Non-sink conditions must be used for observing supersaturation. The degree of supersaturation is also an important factor.
✓ Change in pH during transfer of ASD in the gastrointestinal tract must be considered, especially when solubility of drug or polymer is pH-dependent. Attention to buffer capacity is also required.
✓ If LLPS occurs, attention to free concentration (activity) is required.
✓ The dissolution test is a batch system. Crystallization/LLPS behavior is different if there is an absorption sink, as it decreases degree of supersaturation.
✓ In vivo components, especially bile acids, may influence the supersaturation behavior.

Changes in pH during the transfer of ASD in the gastrointestinal tract must be considered, especially when the solubility of a drug or polymer is pH-dependent. Experience in an acidic environment can significantly alter the dissolution of basic drugs and excipients. The dissolution behavior of acidic components can also be influenced by the experience of an acidic condition. HPMCAS is not soluble in an acidic environment to form insoluble gels that inhibit the release of drugs; thus, the dissolution of the drug is suppressed compared to the simple dissolution test at a neutral pH [15,42]. Attention to buffer species is also required when ionizable components are included in ASDs. Phosphate buffers are typically used for dissolution studies; however, pH in the gastrointestinal tract is maintained by carbonate buffer, which has a lower buffering capacity relative to the phosphate buffer. Thus, the dissolution rate of ionizable components may be overestimated by using phosphate buffer [43]. In the case of HPMCAS, a very low concentration, 5–10 mM of phosphate buffer (pH 6.5), was found to show a “biorelevant” dissolution rate [44].

**Figure 5:**
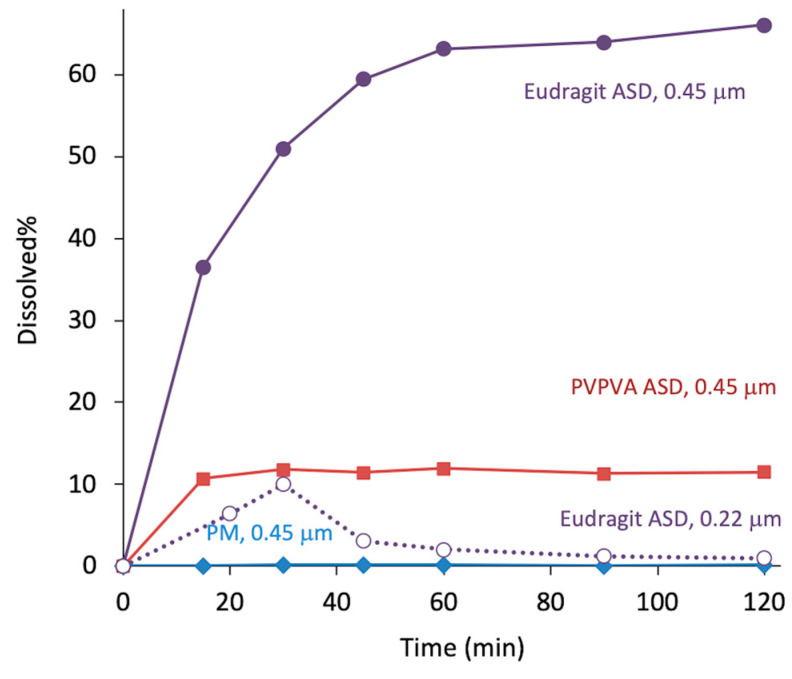
Non-sink dissolution study of MLK ASDs (25 μg/mL as equivalent of MLK). Eudragit L100-55 and PVPVA were used as excipients. All ASDs (MLK:polymer = 1:4) were prepared by freeze-drying. The test solution was filtrated using syringe filters with two different pore sizes (0.22 or 0.45 μm), as noted in the figure. PM represents the physical mixture of crystalline MLK and mannitol. Unpublished results.

When LLPS occurs, the free concentration must be determined to estimate oral absorption, as it is analogous to the activity of the drug. Many in vitro studies have revealed that activity is well-correlated with transmembrane absorption [45,46], although its correlation with in vivo absorption requires consideration of other influential factors [47]. The removal of droplets/particles during the quantification process, which is usually performed by filtration or ultracentrifugation, is sometimes difficult because the size of the LLPS particles is too small to be separated [48,49]. Figure 5 shows the dissolution profiles of montelukast (MLK) ASDs, where two types of syringe filters with different pore sizes were used. When Eudragit L100-55 was used as a polymeric excipient, the LLPS particles passed through the membrane with a 0.45 μm pore, resulting in apparent superior dissolution. However, a dramatic decrease in concentration was found when a syringe filter with a 0.22 μm pore was applied. The apparent LLPS concentrations in the presence of PVPVA, Eudragit, and in the absence of polymers were 2.0, 1.2, and 0.6 μg/mL, respectively. The final concentration of MLK in the presence of PVPVA agreed with the LLPS concentration, whereas those in the presence of Eudragit and in the absence of polymer shifted slightly to lower concentration, presumably because of the presence of excess solids to change equilibrium balance [50]. The evaluation using a 0.22 μm syringe filter offered a good prediction of oral absorption, as shown later.

The dissolution test is a batch system where the dissolved drug is not removed from the test medium throughout the study. As the LLPS/crystallization behavior is influenced by the degree of supersaturation, retention of the drug in the medium, which is different from conditions in the intestinal tract, may accelerate LLPS/crystallization to decrease the free drug concentration. Bevernage et al. revealed that the decrease in the concentration of supersaturated posaconazole solution was slower in the presence of an absorption sink, as the removal of the free drug through the membrane reduced the driving force of crystallization [51]. This result demonstrates the limitations of dissolution studies in predicting the oral absorption of supersaturable dosage forms. To overcome this problem, dissolution tests that include absorption sinks have been proposed, as represented by a biphasic dissolution system [36,52,53]. In this system, the octanol phase is included in the paddle vessel to allow the transfer of the dissolved drug to the octanol phase. This system may work for predicting oral absorption from supersaturable dosage forms to some extent; however, the limitations of this system include the absence of a transfer barrier to the octanol phase and difficulty in including the surface-active component in the medium.

In vivo components may also influence the dissolution process of ASDs. The use of simulated gastric fluids for dissolution studies may work to some extent; however, the composition of the intestinal components is not limited to those in the simulated fluids, and in addition, the inter-individual variance of the intestinal components is substantial [54]. Intestinal components form various types of molecular assemblies, including mixed micelles, vesicles, and oil droplets, which can accommodate lipophilic drugs [54,55,56]. Moreover, surface-active agents can alter the dissolution and supersaturation behaviors of supersaturable formulations [13,14,15,23,45]. Nevertheless, LLPS behaviors in buffer solutions and human intestinal fluids were found to be similar [49].

If a crystalline drug is included in ASDs, even in trace amounts, it can destroy the supersaturation behavior. This is because the remaining crystals may act as templates for crystallization [57,58]. However, it was also observed that a trace amount of crystals did not impact the supersaturation behavior, as they may have dissolved immediately [59,60,61]. Thus, the impact of residual crystal seems to depend on many factors, including the crystallization rate of the drug, the solubility of the drug, and the supersaturation stability of the formulation. Dissolution tests are sometimes more discriminative than other physical characterization methods, including X-ray powder diffraction and differential scanning calorimetry [61]. This means that the dissolution behavior of ASDs can be influenced even without the obvious appearance of crystals in the formulation.

## 4. Membrane Permeability of Supersaturated Drug

Various solubilization technologies are available for poorly soluble drugs. However, the use of solubilization agents may face a solubility–permeability interplay issue [47,62], as an increase in equilibrium solubility using solubilization agents does not increase the activity of the drug. The interplay effect generally does not cause serious issues in vivo [47]; however, it sometimes happens in the presence of strong interactions between the drug and its carrier [63,64]. A great advantage of ASD is that it is free from interplay issues, as it increases the activity of the drug, which can be assessed using membrane permeation studies. The permeation rate is proportional to the difference in the activity of the drug in the donor and acceptor phases, i.e.,

(4) $$\frac{dx_{a}}{dt}=D\left(\gamma_{d}x_{d}-\gamma_{a}x_{a}\right),$$

where *x* and *γ* are the concentration and activity coefficient of the drug, respectively. Subscripts *a* and *d* represent the acceptor and the donor phases, respectively. *t* and *D* denote time and diffusion coefficient, respectively. If the donor phase is saturated with excess solids, *x*_d_ may be replaced with the solubility under the assumption of a sufficiently fast dissolution rate. Thus, if the solution is supersaturated with amorphous solids, *x*_d_ may be replaced by the LLPS concentration, which generates a much stronger driving force for permeation. Moreover, LLPS droplets may be regarded as drug reservoirs that immediately provide lost drug molecules due to permeation.

As discussed in the previous section, the ability of dissolution tests to predict the in vivo absorption is limited. Thus, the observation of membrane permeability provides a greater chance of prediction of oral absorption. When a simple polymeric membrane is used for the test, permeation is dominated by the activity of the drug in the donor phase [47]. If an artificial lipid membrane is used, the drug is distributed to the membrane, in addition to the donor and the acceptor phases. In this situation, the equations to describe the drug distribution are as follows:

(5) $$\frac{dC_{m}}{dt}=k_{d}\left(\frac{{\gamma_{m}S}_{m}}{{\gamma_{d}S}_{d}}-\frac{{\gamma_{m}C}_{m}}{\gamma_{d}C_{d}}\right)$$

(6) $$\frac{dC_{a}}{dt}=k_{a}\left(\frac{\gamma_{m}C_{m}}{{\gamma_{a}C}_{a}}-\frac{{\gamma_{m}S}_{m}}{{\gamma_{a}S}_{a}}\right)$$

Equations (5) and (6) describe the drug distribution from the donor phase to the membrane and from the membrane to the acceptor phase, respectively. Here, *γ*_m_, *S*_m_, and *C*_m_ are the activity coefficient, solubility, and drug concentration in the membrane, respectively. *k*_d_ and *k_a_* are the permeation coefficients into and out of the membrane, respectively. A sufficiently larger *γ*_m_*S*_m_ than *γ*_d_*S*_d_ is required for the effective distribution of the drug from the donor phase to the membrane, whereas an excessively large *γ*_m_*S*_m_ limits the distribution of the drug from the membrane to the acceptor phase. Thus, the composition of the membrane significantly influences permeation results [65]. To facilitate drug release from the membrane to the acceptor phase, solubilization agents must be included in the acceptor phase [66].

**Figure 6:**
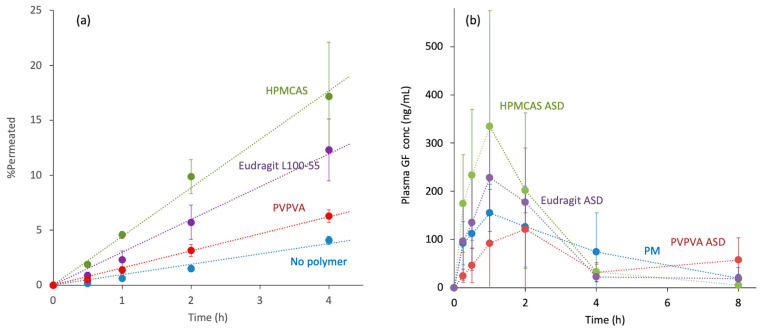
(**a**) The side-by-side membrane permeation study of supersaturated GF solution. The GF concentration in the acceptor phase is presented. An artificial lipid membrane comprising lecithin and dodecane was used for the study. The initial concentrations of GF and polymer in the donor phase were 0.2 mg/mL and 5 mg/mL, respectively. The types of polymers used are indicated in the figure. Unpublished results. (**b**) Plasma GF concentration after administration of GF ASDs to fasted rats at a dose of 10 mg/kg. The polymers used for the ASDs are indicated in the figure. PM indicates the physical mixture of crystalline GF and mannitol. The figures were adopted from ref. [13] after modifications with permission from Elsevier.

A side-by-side cell equipped with an artificial lipid membrane may allow the prediction of oral absorption. Figure 6 shows an example where the oral absorption from GF ASDs was predicted. For the donor phase, GF dissolved in an organic solvent was added to create a supersaturated solution, where various types of polymers were predissolved. The membrane permeability depended on the polymer type (Figure 6a). The results of the oral administration study of GF ASDs are presented in Figure 6b, which exhibited a good correlation with the membrane permeation study.

Many membrane transport studies have suggested that transmembrane flux is dominated by the activity of the drug; however, a detailed investigation suggested that LLPS droplets may also contribute to enhanced drug permeation [66], which was explained by the particle drifting effect [67]. In this explanation, drug droplets/particles are assumed to approach the membrane effectively because they are carried as concentrated droplets/particles. However, the presence of a mucus layer is not ignorable in living systems. Thus, another explanation for the contribution of LLPS droplets may be their effective penetration through the mucus layer to approach the epithelial membrane. In fact, submicron particles are known to diffuse in the mucus layer more rapidly than general thought, depending on their surface properties [68,69].

Surfactants are frequently included in both ASDs and dissolution medium. If the surfactant concentration is above the critical micellar concentration, its negative influence on the membrane permeation of drugs is anticipated [47,62]. Biorelevant media contain large amounts of bile acids that dissolve poorly soluble drugs. Many in vitro membrane permeation studies have revealed that drug molecules dissolved in bile salt micelles cannot permeate through the membrane [46]. However, bile salt micelles are unlikely to inhibit the permeation of the captured drug in vivo [47,70]. Presumably, the inhibitory effect of surfactant micelles depends on the strength of the interaction between the drug and the micelles, which requires further verification.

## 5. Impact of LLPS on Oral Absorption

**Figure 7:**
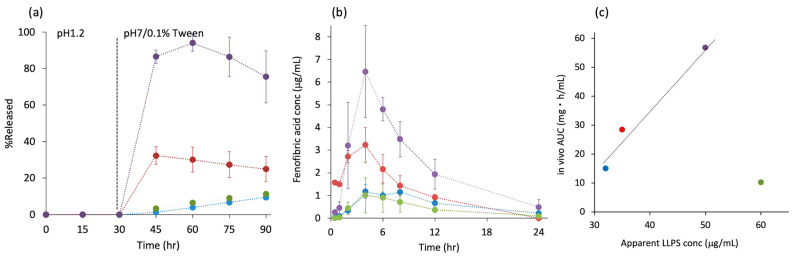
(**a**) pH-shift non-sink dissolution study of FEN ASDs. The test was initiated at pH 1.2, followed by an increase to pH 7.0 at 30 min. Tween 80 was also added at 30 min to reach a concentration of 0.1%. (**b**) Oral administration study of FEN ASDs in fasted rats at a dose of 7.5 mg/kg. Plasma fenofibric acid concentration, which is a major metabolite of FEN, is presented. (**c**) Correlation between apparent LLPS concentration in the presence of polymers and AUC of the oral administration study. Polymer type: Eudragit L100 (purple), PVPVA (red), and HPMCAS (green). The blue symbol represents crystalline FEN. Figures are adopted from ref. [15] after modifications with permission from Elsevier.

ASD technology is usually applied to BCS class II compounds, where the solubility and/or dissolution rate can be limiting factors for oral absorption. As LLPS is analogous to amorphous solubility, LLPS concentration-limited absorption can occur. Figure 7a shows a non-sink dissolution study of fenofibrate (FEN) ASDs [15]. The solubility of FEN does not depend on pH; however, the solubility of Eudragit L100 and HPMCAS does. Thus, a pH shift was required to find a correlation between the dissolution test and the oral administration study. The ASD with Eudragit exhibited the best dissolution, followed by the PVPVA ASD. The HPMCAS ASD failed to improve the dissolution behavior compared with crystalline FEN, as HPMCAS formed a gel during the low pH period, which prevented the release of FEN, even after the addition of Tween. Figure 7b shows the oral absorption of FEN from ASDs in rats [15]. The ASD prepared using Eudragit exhibited the best absorption, followed by the PVPVA ASD. The ASD with HPMCAS did not improve the oral absorption relative to crystalline FEN. This order was in good agreement with that of the dissolution study. Figure 7c shows the relationship between the apparent LLPS concentration of FEN in the presence of each polymer and the area under concentration (AUC) of the plasma fenofibric acid concentration in the rat study [15]. A good correlation was found except for HPMCAS ASD, which indicated the absorption from Eudragit and PVPVA ASDs were solubility (LLPS concentration)-limited. Absorption from the HPMCAS ASD appeared to be limited by the dissolution rate, as this ASD caused gelation during the dissolution process, retarding drug release. As the presence of HPMCAS offered the highest LLPS concentration, if the HPMCAS ASD could be formulated appropriately, e.g., by adding disintegrants, it should provide the best absorption.

**Figure 8:**
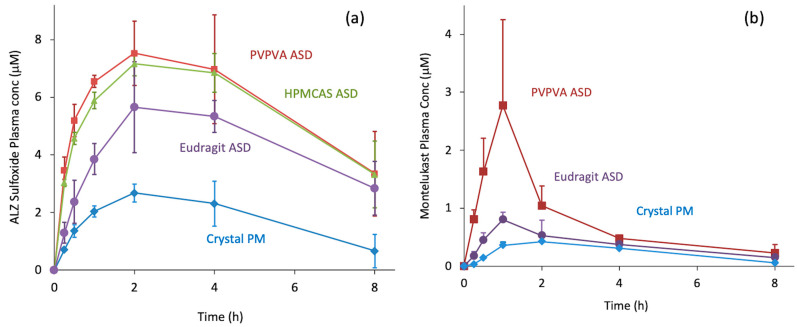
(**a**) Oral administration study of ALZ ASDs and physical mixtures of crystalline ALZ with mannitol in fasted rats at a dose of 10 mg/kg. The polymers used for the ASDs were PVPVA, HPMCAS, and Eudragit L100. The plasma concentration of the major metabolite, ALZ sulfoxide, is presented. The figure was reproduced from ref. [17] with modification under the Creative Commons license. (**b**) Oral administration study of MLK ASDs in fasted rats at a dose of 10 mg/kg. The polymers used were PVPVA and Eudragit L100-55. The ASDs (MLK:polymer = 1:4) were prepared by freeze-drying. The absorption from a physical mixture of crystalline MLK and mannitol is also presented. Unpublished results.

Figure 8 shows other examples where oral absorption could be explained by apparent LLPS concentration [17]. When albendazole (ALZ), a poorly soluble basic drug, was administered in the form of ASDs (Figure 8a), the order of the polymers that offered high exposure was PVPVA, HPMCAS, and Eudragit L100. The apparent LLPS concentrations of ALZ in the presence of these polymers were 7.2, 7.0, and 3.8 μg/mL, respectively. For ALZ in the absence of polymers, the concentration was 1.4 μg/mL. Thus, the order of absorption and apparent LLPS concentrations agreed well. A similar observation was made for MLK ASDs (Figure 8b). MLK is a poorly soluble acidic drug. A significant improvement in oral absorption was observed for the PVPVA ASD, whereas the Eudragit ASD provided only a marginal improvement. This absorption order also agreed well with the apparent LLPS concentrations. The dissolution test (Figure 4) could also predict oral absorption. These observations revealed that the oral absorption from ASDs can be predicted from the apparent LLPS concentration if the absorption is limited by solubility.

**Figure 9:**
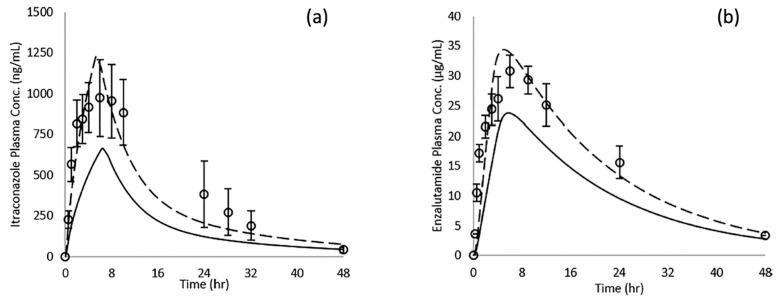
Comparison of model fitting to oral absorption data in rats using GastroPlus^TM^ following conventional procedure (solid lines) and a model which considers particle drifting effect into unstirred water layer (break lines) [71]. (**a**) The fitting to oral absorption of itraconazole from ASD (Sporanox). The absorption data was taken from ref. [72]. (**b**) The fitting to oral absorption of enzalutamide from PVPVA ASD. The absorption data was taken from ref. [73]. Figures are adopted with permission from American Chemical Society. https://pubs.acs.org/doi/10.1021/acs.molpharmaceut.9b00889, accessed on 16 January 2025.

The prediction of plasma concentration profiles after oral administration of ASDs remains challenging. However, an attempt to include the particle drifting effect in conventional prediction protocols has been reported [71]. Figure 9a shows the predicted plasma concentration profile of itraconazole after the oral administration of Sporanox^TM^. By considering the particle drifting effect, the predicted plasma concentrations increased to reach the experimental concentrations. A similar observation was made for the enzalutamide/PVPVA ASD (Figure 9b).

**Figure 10:**
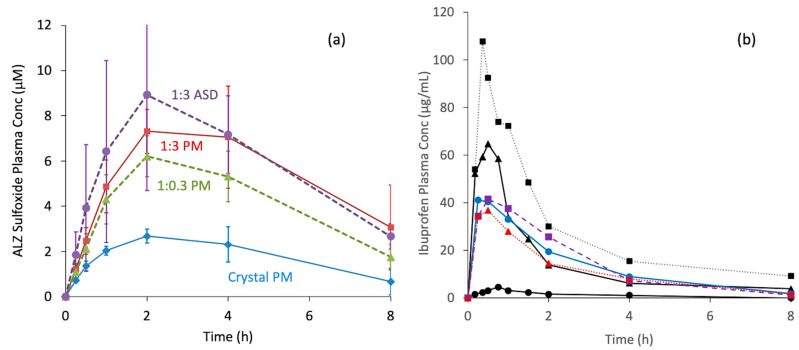
(**a**) Oral administration study of ALZ ASDs and physical mixtures (PM) of crystalline ALZ with Eudragit L100-55 or mannitol (shown as Crystal PM) in fasted rats at a dose of 10 mg/kg. Eudragit L100-55 was used for the ASDs and PMs at the mixing ratio (ALZ:Eudragit) shown in the figure. Unpublished results (except the crystal PM data). (**b**) Oral administration study of IBP ASDs in fasted rats at a dose of 25 mg/kg. The data presented by black symbols are taken from literature with permission from Elsevier, where poloxamer 188 was used as an excipient [74]. The IBP:poloxamer ratios were 2:1 (triangle) or 1:1 (square). The circles represent PM at a ratio of 1:10. Our data is presented by colored symbols. Eudragit L100 (purple squares) or PVP k30 (red triangles) was used as a polymer for ASDs at a mixing ratio of 1:4 (IBP:polymer). The ASDs were prepared by freeze-drying. Blue circles represent crystalline IBP, which had a particle size of a few μm. The error bars were omitted for clarity, but the deviations were significantly large for the literature data on ASDs. Our data are unpublished results.

Numerous studies that claimed the enhancement of oral absorption using ASD technology are available in the literature. However, the use of the amorphous state may not have been a key factor in some cases. Basic drugs can be supersaturated if they are transferred from an acidic to a neutral pH. Therefore, amorphization may not be required to design supersaturable dosage forms. Figure 10a shows the oral absorption of ALZ from ASDs and physical mixtures (PMs) with an identical composition. Absorption from the PMs significantly increased with the addition of Eudragit L100-55 as an excipient. The absorption of ALZ from the 1:3 PM was slightly lower but almost identical to that from the 1:3 ASD, suggesting that the supersaturation–maintenance effect of the polymer was likely to be more important than the dissolution enhancement by amorphization. The absorption enhancement was found to depend on the amount of the polymer added. This observation indicated that the oral absorption of basic drugs can be improved without amorphization if a polymer that can maintain the supersaturation is used.

Another example presented below is a case where the evaluation of the reference crystal was inappropriate (Figure 10b). In the literature, the oral absorption of ibuprofen has been reported to be improved by using ASD technology, where poloxamer 188 was used as an excipient [74]. Absorption from the crystalline PM was extremely low in this study. Our similar attempt using ASDs yielded similar plasma concentration profiles as shown in the figure. However, as the absorption from crystalline PM was similar to that from ASDs, we could not find any improvement in absorption using ASDs. The difference between our study and the literature is the difference in the absorption from crystalline PM. The most likely reason for this is the difference in the particle sizes of the IBP crystals, as absorption of poorly soluble drugs can be limited by the dissolution rate if the particle size is too large. IBP does not require amorphization to improve its oral absorption.

## 6. Conclusions

Recently updated knowledge on the dissolution, membrane permeation, and oral absorption behaviors of ASDs is presented with a focus on LLPS behavior. As the dissolution process is sensitive to various factors, including the applied dose, medium composition, and pH, these factors must be considered when designing a dissolution test for predicting in vivo absorption. Supersaturated solutions can cause LLPS and/or crystallization. The LLPS concentration, which is analogous to the amorphous solubility, is typically higher than the crystalline solubility by more than an order of magnitude. Transmembrane flux is governed not by apparent solubility but by activity. Therefore, ASD has an advantage over other solubilization techniques that increase the equilibrium solubility to increase the flux. Moreover, LLPS droplets are expected to increase the transmembrane flux by increasing the drug concentration near the epithelial cell membrane, possibly through the particle drifting effect and/or effective permeation through the mucus layer. The LLPS concentration can be a dominant factor in predicting oral absorption from ASDs if absorption is limited by solubility. A model prediction that considers the particle drifting effect in the unstirred water layer is also available. An appropriate formulation design is necessary for finding these advantages of ASDs.

## Figures and Tables

**Table 1:** Equilibrium crystalline solubility and LLPS concentrations of poorly soluble drugs.

Compound	Temperature	pH	Crystalline Solubility (μg/mL)	LLPS Conc. (μg/mL)	LLPS/Crystal Solubility Ratio	Reference
Albendazole	25	7.0	< 0.1	1.4	>14	[17]
Clotrimazole	37	10.0	0.4	5.2	13	[18]
Clozapine	37	10.0	8.8	136	15	[18]
Danazol	25	6.8	0.9	13	14	[19]
Diclofenac sodium	37	1.2	3.4	92	27	[20]
Efavirenz	37	6.8	8.2	18.4	2.2	[18]
Felodipine	37	6.8	0.94	9.8	10	[18]
Fenofibrate	25	7.0	0.1	1.0	10	[15]
Griseofulvin	37	7.0	12	38	3.2	[13]
Indomethacin	37	2.0	3.0	30.4	10	[18]
Ketoconazole	37	10.0	3.7	54.4	15	[18]
Loratadine	37	6.8	1.6	7.6	4.8	[18]
Nifedipine	37	6.8	1.4	45	32	[21]
Naftopidil	37	6.8	10.0	58.3	5.8	[22]
Posaconazole	37	6.5	1.7	12	7.1	[23]
Ritonavir	37	6.8	1.3	18.8	14	[18]

## Data Availability

The raw data supporting the conclusions of this article will be made available by the authors upon request.
